# Supplementary material for: Most long-lived contrails form within cirrus clouds with uncertain climate impact
Source: Nat Commun. 2025 Nov 3;16:9695. doi: 10.1038/s41467-025-65532-2 (PMC12583729; doi:10.1038/s41467-025-65532-2)
Supplement: Supplementary file 1 — Supplementary Information [file 41467_2025_65532_MOESM1_ESM.pdf]

Most long-lived contrails form within cirrus clouds with uncertain climate impact – Supplementary information

Andreas Petzold[1, 2], Neelam F. Khan[1,3], Yun Li[1], Susanne Rohs[1], Susanne Crewell[3], Andreas Wahner[1], and Martina Krämer[4, 5]

[1] Institute of Climate and Energy Systems 3 – Troposphere, Forschungszentrum Jülich GmbH, Jülich, Germany
[2] Institute for Atmospheric and Environmental Research, University of Wuppertal, Wuppertal, Germany
[3] Institute for Geophysics and Meteorology, University of Cologne, Cologne, Germany
[4] Institute of Climate and Energy Systems 4 – Stratosphere, Forschungszentrum Jülich GmbH, Jülich, Germany
[5] Institute for Atmospheric Physics, Johannes Gutenberg-University Mainz, Mainz, Germany

Correspondence to: Andreas Petzold (a.petzold@fz-juelich.de)

Content

1 Supplementary Figures 2

2 Supplementary Tables 4

1 Supplementary Figures

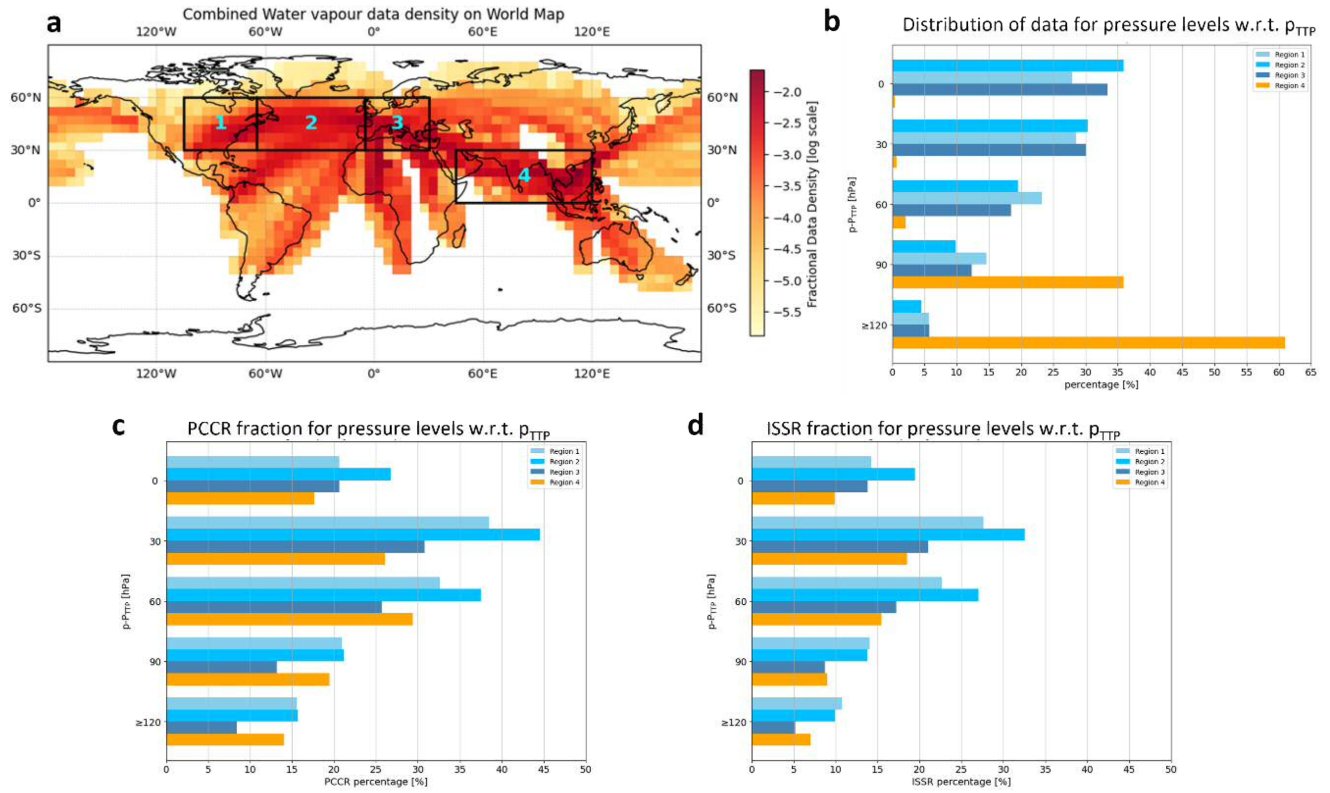

Figure S1. Global coverage of IAGOS water vapour data for the period 2014 – 2021, including regional resolution for the Northern Midlatitudes. Panel (a): Fractional data density of IAGOS combined water vapor dataset from June 2014 to December 2021 (logarithmic scale used to visualize maximum data coverage); inserted boxes indicate the regions of interest Eastern North America (Region 1), the North Atlantic (Region 2), Western Europe (Region 3) and Southeast Asian Subtropics (Region 4); Panel (b): vertical distribution of data for four pressure levels of thickness 30 hPa relative to the pressure level of the thermal tropopause p_{TTP}; Panel (c) fraction of potential contrail-cirrus regions (PCCR; $RH_{ice} \geq 90\%$) for each pressure level; Panel (d) same as Panel (c) but for ice-supersaturated regions (ISSR; $RH_{ice} \geq 100\%$).

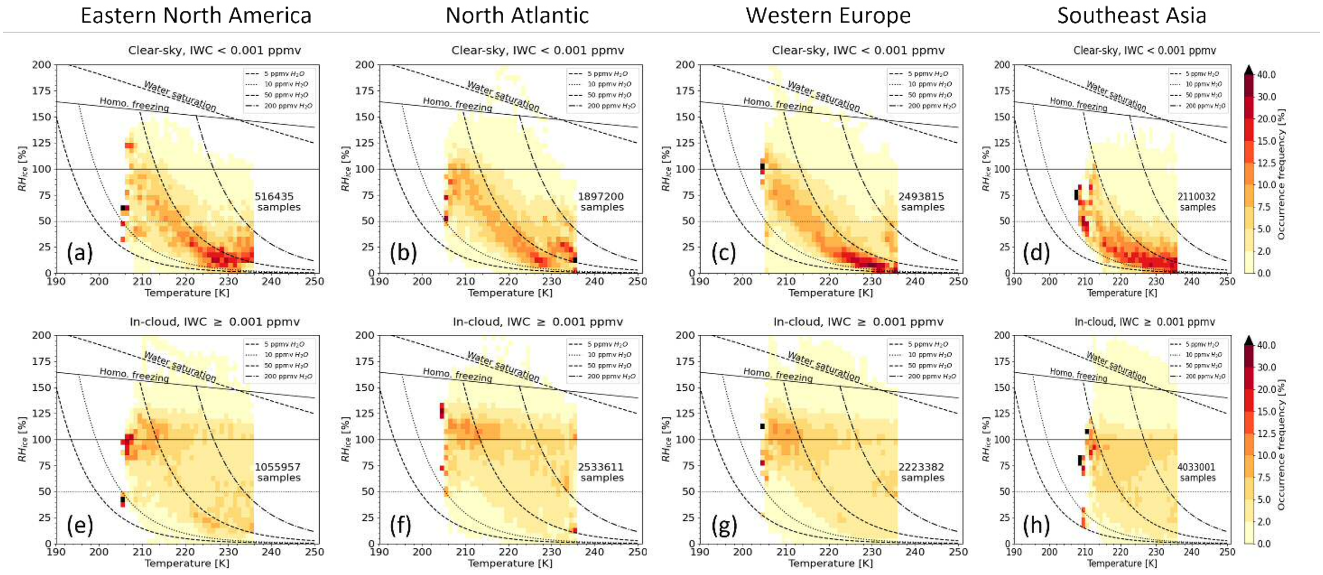

Figure S2. Observed distributions of relative humidity with respect to ice (RH_{ice}) for clear-sky and in-cloud conditions as function of temperature. RHI_{ice} for clear-sky ((a) – (d)) and in-cloud ((e) - (h)) conditions in the respective regions; the classification into clear-sky and in-cloud used the ERA5 CIWC - based cloud index; data were analysed relative to the thermal tropopause TTP for $p < 350$ hPa $< p_{TTP}$ – 15 hPa.

The influence of air temperature on the distribution of RH_{ice} inside clouds and in clear sky is further analysed in S2. Here, we sorted the RH_{ice} data into temperature bins of 1 K width and calculated the occurrence distribution per temperature bin. The iso-lines shown in S2 correspond to lines of constant water vapour volume mixing ratio as function of temperature. For clear-sky conditions (S2a to d), the most frequently observed RH_{ice} values represent dry conditions with RH_{ice} < 25% at temperatures above 225 K, while the coldest air masses probed at temperatures below 215 K are frequently close to ice-saturation. It should be noted that the latter conditions were sampled only at midlatitudes (S2a to c), whereas in the subtropics (S2d), air temperatures below 215 K are sparse, and ice-saturation is rarely reached. Inside cirrus clouds at midlatitudes (S2e - g), most frequent RH_{ice} values scatter around ice-saturation with a slight tendency towards ice-supersaturation up to 120% at the colder temperatures below 215 K. For the subtropics (S2h), the most frequent RH_{ice} values are found slightly below ice-saturation, independent of the air temperature.

2 Supplementary Tables

Table S1. Fractions of clear-sky and in-cloud areas for the analysed regions with the respective status of contrail existence added, for in-istu observations and ERA5 model fields; regions are Eastern North America (Region 1), the North Atlantic (Region 2), Western Europe (Region 3) and Southeast Asian Subtropics. Potential contrail-cirrus regions are characterised by a threshold of relative humidity with respect to ice (RH_{ice}) for contrail-cirrus existence of 90%, and ice-supersaturated regions by a threshold value for RH_{ice} of 100%.

		**Potential Contrail-Cirrus Regions**						**Ice-Supersaturated Regions**					
			clear sky			in-cloud			clear sky			in-cloud	
Northern Midlatitudes	Total no. samples		4907450			5812950			4907450			5812950	
	Fraction of total, %		45.78			54.22			45.78			54.22	
	Contrail status	no	short-lived	long-lived	no	short-lived	long-lived	no	short-lived	long-lived	no	short-lived	long-lived
	No. sub-samples	73876	4438217	395357	66872	3157228	2588850	73876	4632652	200922	66872	3854435	1891643
	Fraction of total, %	0.7	41.4	3.7	0.6	29.5	24.2	0.7	43.2	1.9	0.6	36.0	17.7
Southeast Asian Subtropics	Total no. samples		2110032			4033001			2110032			4033001	
	Fraction of total, %		34.34			65.65			34.34			65.65	
	Contrail status	no	short-lived	long-lived	no	short-lived	long-lived	no	short-lived	long-lived	no	short-lived	long-lived
	No. sub-samples	299869	1795636	14527	593448	2588775	850778	299869	1804491	5672	593448	3022954	416599
	Fraction of total, %	4.9	29.2	0.2	9.7	42.1	13.9	4.9	29.4	0.1	9.7	49.2	6.8
Eastern North America	Total no. samples		516435			1055957			516435			1055957	
	Fraction of total, %		32.84			67.15			32.84			67.15	
	Contrail status	no	short-lived	long-lived	no	short-lived	long-lived	no	short-lived	long-lived	no	short-lived	long-lived
	No. sub-samples	7303	478645	30487	20629	625719	409609	7303	494626	14506	20629	741278	294050
	Fraction of total, %	0.5	30.4	1.9	1.3	39.8	26.1	0.5	31.5	0.9	1.3	47.1	18.7
North Atlantic	Total no. samples		1897200			2533611			1897200			2533611	
	Fraction of total, %		42.81			57.18			42.81			57.18	
	Contrail status	no	short-lived	long-lived	no	short-lived	long-lived	no	short-lived	long-lived	no	short-lived	long-lived
	No. sub-samples	14454	1697360	185386	13782	1238696	1281133	14454	1788430	94316	13782	1556229	963600
	Fraction of total, %	0.3	38.3	4.2	0.3	28.0	28.9	0.3	40.4	2.1	0.3	35.1	21.7
Western Europe	Total no. samples		2493815			2223382			2493815			2223382	
	Fraction of total, %		52.86			47.13			52.86			47.13	
	Contrail status	no	short-lived	long-lived	no	short-lived	long-lived	no	short-lived	long-lived	no	short-lived	long-lived
	No. sub-samples	52119	2262212	179484	32461	1292813	898108	52119	2349596	92100	32461	1556928	633993
	Fraction of total, %	1.1	48.0	3.8	0.7	27.4	19.0	1.1	50.0	2.0	0.7	33.0	13.4

Table S2. Regional split of coverage areas with Schmidt-Appleman criterion (SAC) fulfilled and further subdivided into classes of potential climate impact for areas with SAC fulfilled, and respective contrail-cirrus overlap; data are based on IAGOS in-situ RH_{ice} observations, SAC from T observations, and ERA5 cloud ice water content (CIWC) cloud categorisation; regions are Northern Midlatitudes, Southeast Asian Subtropics, Eastern North America, the North Atlantic, and Western Europe.

Region	Threshold RH_{ice}	Fraction of potential contrail-cirrus areas with SAC fulfilled				Climate impact			Contrail – cirrus overlap CIWC visibility threshold	
		clear sky CIWC < 0.001 ppmv	subvisible cirrus 0.001 ppmv ≤ CIWC < 1.0 ppmv (2.0 ppmv)	visible cirrus CIWC ≥ 1.0 ppmv (2.0 ppmv)	total	warming	ambiguous	no	1.0 ppmv	2.0 ppmv
column		a	b	c	a + b + c	a + b	c	100 – (a + b + c)	100 c / (a + b + c)	
Northern Midlatitudes	PCCR (≥ 90%)	3.7%	9.0% (11.5%)	15.2% (12.8%)	28.0%	12.7% (15.1%)	15.2% (12.8%)	72.1%	54.5%	45.7%
	ISSR (≥ 100%)	1.9%	5.9% (7.6%)	11.8% (10.1%)	19.6%	7.8% (9.5%)	11.8% (10.1%)	80.4%	60.2%	51.5%
SE Asian Subtropics	PCCR	0.2%	1.1% (1.6%)	12.7% (12.2%)	14.0%	1.4% (1.9%)	12.7% (12.2%)	85.9%	90.7%	87.1%
	ISSR	0.1%	0.5% (0.8%)	6.3% (6.0%)	6.9%	0.6% (0.9%)	6.3% (6.0%)	93.1%	91.3%	87.0%
Eastern North America	PCCR	1.9%	7.1% (9.4)	19.2% (17.0%)	28.3%	9.1% (11.3%)	19.2 (17.0%)	71.7%	67.8%	60.0%
	ISSR	0.9%	4.3% (5.8%)	14.6% (13.1%)	19.8%	5.2% (6.7%)	14.6% (13.1%)	80.2%	73.7%	66.2%
North Atlantic	PCCR	4.2%	10.7% (13.8%)	18.3% (15.2%)	33.2%	14.9 (18.0%)	18.3% (15.2%)	66.8%	55.1%	45.8%
	ISSR	2.1%	7.1% (9.4%)	14.7% (12.4%)	23.9%	9.2% (11.5%)	14.7% (12.4%)	76.1%	61.5%	51.9%
Western WEurope	PCCR	3.8%	8.1% (10.0%)	11.1% (9.1%)	23.0%	11.9% (13.8%)	11.1% (9.1%)	77.0%	48.3%	39.6%
	ISSR	2.0%	5.3% (6.6%)	8.3% (6.9%)	15.5%	7.2% (8.6%)	8.3% (6.9%)	84.5%	53.5%	44.5%
